# Splenic T2 signal intensity loss on MRI is associated with disease burden in multiple myeloma

**DOI:** 10.1007/s00330-024-11191-8

**Published:** 2024-11-27

**Authors:** Christian Neelsen, Christos Sachpekidis, Lukas John, Peter Neher, Elias Mai, Martin Grözinger, Daniel Paech, Antonia Dimitrakopoulou-Strauss, Felix T. Kurz, Sandra Sauer, Marc S. Raab, Heinz-Peter Schlemmer, Markus Wennmann, Niels Weinhold

**Affiliations:** 1 German Cancer Research Center, Division of Radiology, Heidelberg, Germany; 2 German Cancer Research Center, Clinical Cooperation Unit Nuclear Medicine, Heidelberg, Germany; 3 Department of Medicine V, Multiple Myeloma Section, University Hospital Heidelberg, Heidelberg, Germany; 4 Medical Image Computing, German Cancer Research Center, Heidelberg, Germany; 5 Department of Radiology, Brigham and Women’s Hospital, Harvard Medical School, Boston, MA US

**Keywords:** Multiple myeloma, Neoplasm staging, Multiparametric magnetic resonance imaging, Spleen, Positron emission tomography computed tomography

## Abstract

**Objectives:**

This study aims to evaluate correlations between spleen signal changes in different MRI sequences and bone marrow plasma cell infiltration as potential indicator of disease burden in multiple myeloma (MM) patients.

**Materials and methods:**

We retrospectively analyzed 45 patients with newly diagnosed MM that underwent whole-body MRI with axial DWI at b-values 50 (b50) and 800 (b800), and coronal T1 and T2 fast spin-echo (T2-TSE) imaging. A subcohort of 39 patients had concomitant [^18^F]FDG PET/CT. The spleen was segmented in all MRI sequences and signal intensities were normalized. MR signal intensities and ADC values were correlated with bone marrow plasma cell infiltration from biopsy, laboratory markers (Beta 2-microglobulin, M-Protein, Red blood count (RBC), Hemoglobin, Hematocrit, Total protein, Creatinine), clinical data (ISS stages, high-risk chromosomal aberrations), and standardized uptake value (SUV) in the spleen as well as spleen-to-liver and spleen-to-blood pool SUV ratios on [^18^F]FDG PET-CT.

**Results:**

Bone marrow plasma cell infiltration was negatively correlated with (normalized) mean splenic signal intensity on DWI-b50, DWI-b800, and T2-TSE images (*r* = −0.64, *p* < 0.001, *r* = −0.58, *p* < 0.001, and *r* = −0.66, *p* < 0.001, respectively) while there was no correlation with the apparent diffusion coefficient or spleen size (*p* = 0.52). In the subgroup analysis of 39 patients with concomitant [^18^F]FDG PET-CT, there was no correlation of normalized splenic [^18^F]FDG uptake either with MR spleen signal (for T2 *p* = 0.64) or with bone marrow plasma cell infiltration (*p* = 0.37).

**Conclusions:**

Our findings reveal a significant association between spleen signal intensity especially on normalized T2-weighted images and tumor burden.

**Key Points:**

***Question*** *What changes occur in spleen signal on MRI as tumor load marker changes in multiple myeloma (MM)?*

***Findings*** *Spleen signal intensity, particularly on T2-weighted MRI, negatively correlates with bone marrow plasma cell infiltration and laboratory markers of tumor burden.*

***Clinical relevance*** *Standardized quantification of splenic T2 signal is proposed as a new marker for MM disease burden.*

**Graphical Abstract:**

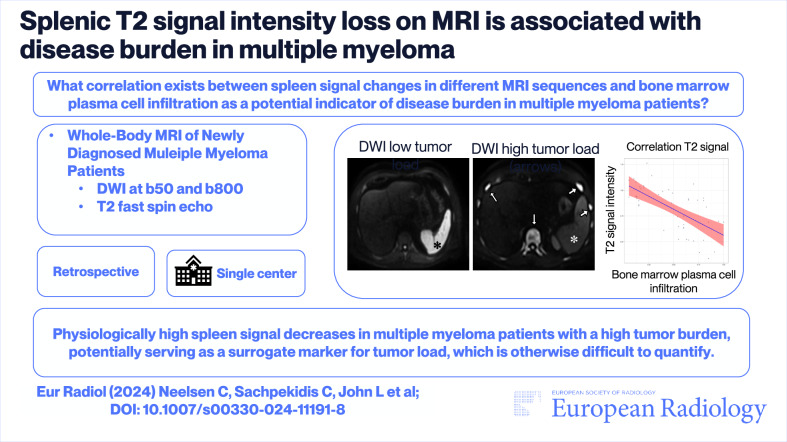

## Introduction

Multiple myeloma (MM) is a neoplastic disorder characterized by monoclonal plasma cell expansion in the bone marrow [1]. While MM is considered incurable, a substantial subset of patients can achieve long-term remissions and survival [2].

Staging in MM encompasses the evaluation of prognostic genetic markers and the assessment of tumor burden [3]. It is well-established that a higher tumor burden is associated with poor prognosis [2]. However, accurately quantifying tumor burden presents a considerable challenge due to the heterogeneous distribution of the disease [4–6] and the well-recognized limitations associated with laboratory markers [2], biopsy procedures [7], and imaging techniques [8].

Recently, loss of spleen signal on diffusion-weighted MRI (DWI) has been linked to tumor burden and worse prognosis in MM, while the underlying mechanism remains unclear [9, 10]. However, these studies focused exclusively on signal intensity on high b-value DWI and did not incorporate quantitative Apparent Diffusion Coefficient (ADC) values or other imaging sequences such as T1 or T2, which could provide additional insights. Furthermore, the biological mechanisms underlying these findings have not yet been fully elucidated [9, 10]. In this retrospective analysis, our objectives are (1) to investigate whether this association is linked to T2 relaxation effects, aiming to identify an objective and reproducible imaging biomarker for an overall assessment of tumor burden; (2) to determine the most effective imaging sequences for customizing imaging protocols; and (3) to explore potential implications of the spleen in myeloma-related processes that remain elusive, utilizing an integrated approach involving multiparametric MRI and PET-CT.

## Materials and methods

### Cohort

We conducted a retrospective analysis of patients who underwent MRI and bone marrow biopsy at our institution, with prior written informed consent from the German-Speaking Myeloma Multicenter Group (GMMG)-HD7 trial (EudraCT: 2017-004768-37). A subgroup of these patients also underwent concomitant whole-body [^18^F]FDG PET/CT. The study was approved by the institutional review board (Approval IDs: S278-13 and AFmu-412/2018 for the biopsy and imaging studies, respectively) and the Federal Agency of Radiation Protection in Germany (“Bundesamt für Strahlenschutz”) and conducted in accordance with the Declaration of Helsinki.

General inclusion criteria of the GMMG HD7 trial included patients aged between 18 and 70 years with newly diagnosed multiple myeloma (NDMM) and abnormal monoclonal protein in serum or urine. Another prerequisite was eligibility for high-dose therapy with autologous stem cell transplantation. Exclusion criteria for the GMMG HD7 trial were systemic AL amyloidosis, plasma cell leukemia, and previous radio- or chemotherapy. From this cohort, the current analysis included all patients who received a baseline 1.5-T MRI scan at the German Cancer Research Center and from whom both biopsy results and all standard MRI sequences (T1, T2, B50, B800, and ADC) were available in sufficient quality. One patient had to be excluded due to severely degraded image quality (zipper artifact most likely due to RF interference).

**Fig. 1:**
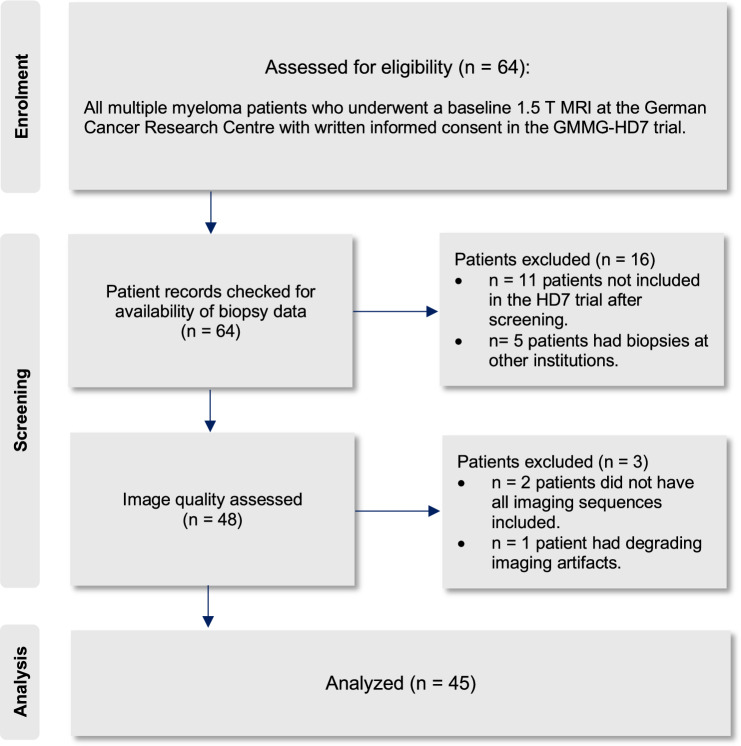
Patient inclusion and exclusion flow diagram

In total, 45 patients qualified for the final analysis (Table 1, Fig. 1). All images were acquired between November 2018 and September 2020.

**Table 1:** Study participants’ characteristics

Characteristics		Median [range]
Age (years)		61 [39–88]
BM plasma cell infiltration (%)		54% [0–100%]
Myeloma protein (g/L)		33.8 [6.2–78.6]
SFLC-ratio		80.2 [1.4–1811]
Calcium (mmol/L)		2.32 [2.94–2.07]
Hemoglobin (g/dL)		12.2 [14.9–8.1]
Creatinine (mg/dL)		0.84 [0.58–3.45]

Characteristics		Number of patients (*n*)
Sex (m:f)		34:11
ISS stage I (*n*)		26
ISS stage II (*n*)		13
ISS stage III (*n*)		6
R-ISS stage I (*n*)		18
R-ISS stage II (*n*)		25
R-ISS stage III (*n*)		2
Beta 2-microglobulin (*n*)	≤ 3.5 mg/L	32
	≥ 5.5 mg/L	6
Albumin < 3.5 g/dL (*n*)		7
Abnormal LDH (*n*)		1
High risk del (17p), t (4; 14), t (14; 16) (*n*)		13

For the assessment of tumor load, we used bone marrow (BM) plasma cell infiltration (PCI) on biopsy as well as the following laboratory markers as a surrogate: Beta 2-microglobulin, M-Protein, total protein, Red blood count (RBC), Hemoglobin, Hematocrit, and Creatinine. Bone marrow (BM) plasma cell infiltration (PCI) was defined as the maximum of both BM aspirate and BM trephine biopsy in accordance with IMWG diagnostic criteria [11]. The average time between bone marrow biopsy and MRI was 7.9 days (median 5, range [0, 29] days).

### Image acquisition and protocol

#### MRI

All patients were examined on a 1.5-T Siemens Magnetom Aera MRI scanner (Syngo MR E11, Siemens Healthineers). A standardized whole-body MRI protocol conforming to current state-of-the-art practice in multiple myeloma was used for clinical relevance [12]. The study protocol encompassed coronal T1 TSE, coronal T2 TSE STIR, and axial DWI at b-values 50 (b50) and 800 (b800) (see Supplementary Table S1 with previously published imaging parameters [13]).

#### PET-CT

A subgroup of 39 patients underwent concomitant whole-body [^18^F]FDG PET/CT. Imaging was performed approximately 60 min post-injection (p.i.) of [^18^F]FDG from the skull to the toes with an image duration of 2 min per bed position. A dedicated PET/CT system (Biograph mCT, S128, Siemens Healthineers) with an axial field of view of 21.6 cm with TruePoint and TrueV, operated in a three-dimensional mode, was used. A low-dose attenuation CT (120 kV, 30 eff mA) was used for attenuation correction of the PET data and for image fusion. All PET images were attenuation-corrected, and an image matrix of 400 × 400 pixels was used for iterative image reconstruction. Iterative image reconstruction was based on the ordered subset expectation maximization (OSEM) algorithm with two iterations and 21 subsets, as well as time of flight (TOF).

### Image analysis

The whole spleen was manually segmented in all sequences (T1, T2, and DWI) by a radiology attending and resident with 10 and 3 years’ experience in body MRI, respectively, using the Medical Imaging Interaction Toolkit, Version 2022.10 [14]. Readers were blinded to the clinical and pathological data.

**Fig. 2:**
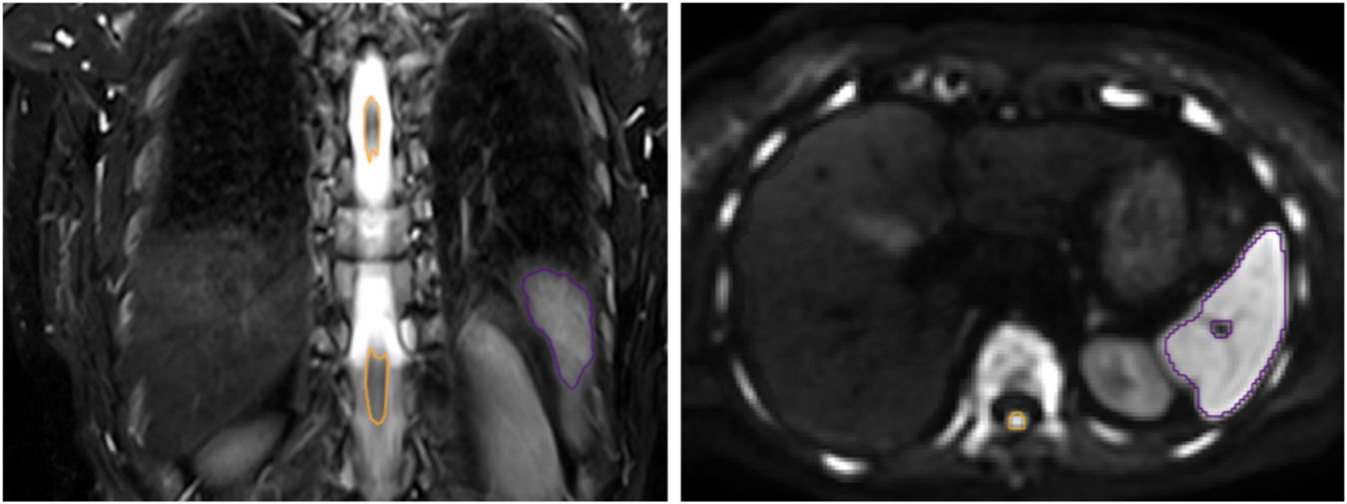
Examples of segmentations of the spleen (violet) and the spinal cord (orange) on coronal T2-TSE (right) and axial DWI (b800, left)

Although all patients were examined with the same scanner hardware as well as the same scan parameters, we performed an additional analysis using normalized DWI and T2-weighted images to adjust for shim and patient factors. The spinal cord served as reference tissue providing a spleen-to spinal cord ratio as proposed by Terao et al [10]. The reasoning was that the spinal cord is protected by the blood-brain barrier and should not be affected by MM-related changes [10]. Therefore, the spinal cord was segmented on DWI and T2-TSE. Coronary T2-TSE was normalized by the average signal intensity of the spinal cord. Axial DWI used slice-specific dynamic shimming (iShim). Therefore, we used a slice-wise normalization, dividing by the signal intensity of the spinal cord of each individual slice. Example segmentations of the spleen and the spinal cord are shown in Fig. 2. T1, T2, b50 and b800 signal intensities before and after normalization, as well as ADC values, were extracted.

[^18^F]FDG PET-CT analysis consisted of calculations of standardized uptake value (SUV) in the spleen and in reference organs, namely the liver and the blood pool, by two experienced, board-certified nuclear medicine physicians with 11 and 30 years’ clinical experience in MM PET diagnostics. In particular, the SUV_mean_ and SUV_max_ of the spleen parenchyma, the liver parenchyma and the blood pool were measured after placing a volume of interest (VOI) in the central part of the spleen, on the right liver lobe as well as in the lumen of the descending aorta in the mediastinum without inclusion of the aortic wall, respectively [15, 16]. On the basis of these measurements, the spleen-to-liver SUV ratios (SLR_mean_, SLR_max_) and spleen-to-blood pool SUV ratios (SBR_mean_, SBR_max_) were calculated. VOIs were drawn using the pseudo-snake algorithm of the Pmod software [17].

### Statistical analysis

Interreader reproducibility of segmentations was assessed using the Sørensen-Dice coefficient [18], while the Intraclass correlation coefficient ICC(A,1) [19] was utilized for the resulting signal intensity measurements. Pearson’s correlation coefficient (*r*) was employed to explore the associations between image intensity values and tumor load parameters (Table 2). Where values did not satisfy normal distribution according to the Shapiro-Wilk test (α = 0. 5) marked with † in the results [20], a bias-corrected and accelerated bootstrap with 1000 samples was applied to calculate confidence intervals [21]. Differences between correlations were tested for significance with Meng’s *z* for correlated correlations [22]. All statistics were performed in R version 4.2.1 (2022-06-23).

**Table 2:** Pearson’s correlation of mean signal intensity with plasma cell infiltration (Pearson's *r* [confidence interval], *p*-value)

Parameter	Plasma cell infiltration (PCI) (*n* = 45)
T2-TSE-	−0.63 [−0.75, −0.45]† *p* < 0.001***
Normalized T2-TSE	−0.66 [−0.77, −0.51]† *p* < 0.001***
B50	−0.61 [−0.74, −0.39]† *p* < 0.001***
Normalized B50	−0.64 [−0.76, −0.45]† *p* < 0.001***
B800	−0.61 [−0.75, −0.35]† *p* < 0.001***
Normalized B800	−0.58 [−0.72, −0.35]† *p* < 0.001***
T1-TSE	−0.37 [−0.57, −0.13]† *p* = 0.013*
ADC (µm^2^/s)	−0.18 [−0.42, 0.07]† *p* = 0.235
Volume (mm^3^)	0.06 [−0.19, 0.36]† *p* = 0.686

Confidence intervals are shown in parentheses

† A bias-corrected and accelerated bootstrap with 1000 samples was applied

* Correlation is significant at the 0.05 level (2-tailed)

*** Correlation is significant at the 0.001 level (2-tailed)

## Results

### Correlation between splenic MRI signal intensity and bone marrow biopsy

Excellent interreader reproducibility was found for both T2-TSE as well as DWI segmentations with Sørensen-Dice coefficient (Dice_T2-TSE_ = 0.926 for and Dice_DWI_ = 0.932) as well as for the resulting signal intensity values assessed by Intraclass correlation coefficient (ICC), ICC_T2-TSE_ = 0.999, *p* < 0.001, CI 0.999–1 and ICC_b50_ = 0.998, *p* < 0.001, CI 0.997–0.999 (corresponding Bland-Altman plots can be found in the Supplementary Fig. 1).

Next, we correlated imaging data with clinical parameters. We observed a negative correlation between signal intensity on high b-value DWI (b800) and disease burden measured by PCI (*r* = 0.61, *p* < 0.001) and laboratory activity markers such as ß-2 microglobulin (*r* = 0.48, *p* = 0.001), and M protein (*r* = 0.49, *p* = 0.001), confirming recent observations [9, 10].

Yet, the same effect was observed on T2-weighted imaging sequences (Table 3; T2-TSE *r* = −0.63 and DWI-b50 *r* = −0.61, *p* < 0.001). There was also a smaller correlation with splenic signal intensity on T1 (*r* = −0.37, *p* = 0.01), however, the correlations were significantly more robust on T2 (Meng’s *z* = 2.41, *p* = 0.008) and Diffusion-weighted imaging (with *z* = 1.75, *p* = 0.041). In contrast, there was no significant correlation between tumor load parameters and spleen size (*p* = 0.69) or the apparent diffusion coefficient (*p* = 0.24).

**Table 3:** Pearson’s correlation of signal intensity on normalized T2-TSE with laboratory markers

Laboratory parameter	Pearson’s *r*, *p*-value
Beta 2-microglobulin (*n* = 45)	−0.50 [−0.65, −0.03]†, *p* = 0.001***
M-Protein (*n* = 40)	−0.46 [−0.67, −0.17], *p* = 0.003**
Red blood count (*n* = 45)	0.49 [0.23, 0.69], *p* = 0.001***
Hemoglobin (*n* = 45)	0.42 [0.14, 0.64], *p* = 0.004**
Hematocrit (*n* = 45)	0.43 [0.15, 0.64], *p* = 0.004**
Total protein (*n* = 44)	−0.39 [−0.62, −0.11], *p* = 0.008**
Creatinine (*n* = 45)	−0.28 [−0.54, 0.22]†, *p* = 0.066

Confidence intervals are shown in parentheses

† A bias-corrected and accelerated bootstrap with 1000 samples was applied

** Correlation is significant at the 0.01 level (2-tailed)

*** Correlation is significant at the 0.001 level (2-tailed)

**Fig. 3:**
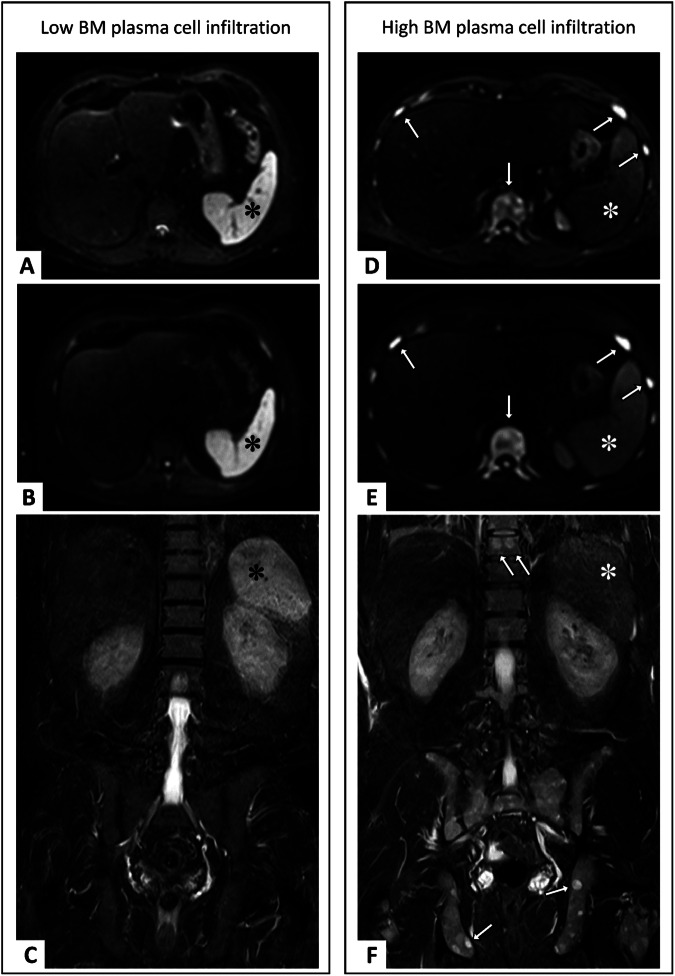
Example of two cases with different levels of bone marrow plasma cell infiltration with identical window leveling correspondingly. On the left, a physiologically hyperintense spleen (✽) is seen on axial DWI-b50 (**A**), -b800 (**B**) and coronary T2 TSE (**C**) in a patient with low bone marrow plasma cell infiltration (17%). On the right, there is significant loss of spleen signal (✽) on intrinsically T2-weighted b50 (**D**), b800 (**E**), as well as T2-TSE (**F**) images in a patient with high bone marrow plasma cell infiltration (85%). On the right, in addition, diffuse increased bone marrow signal on DWI (arrows on **D** and **E**), as well as multiple focal lesions in the pelvis and spine on coronal T2 images (arrows on **F**), can be noted

**Fig. 4:**
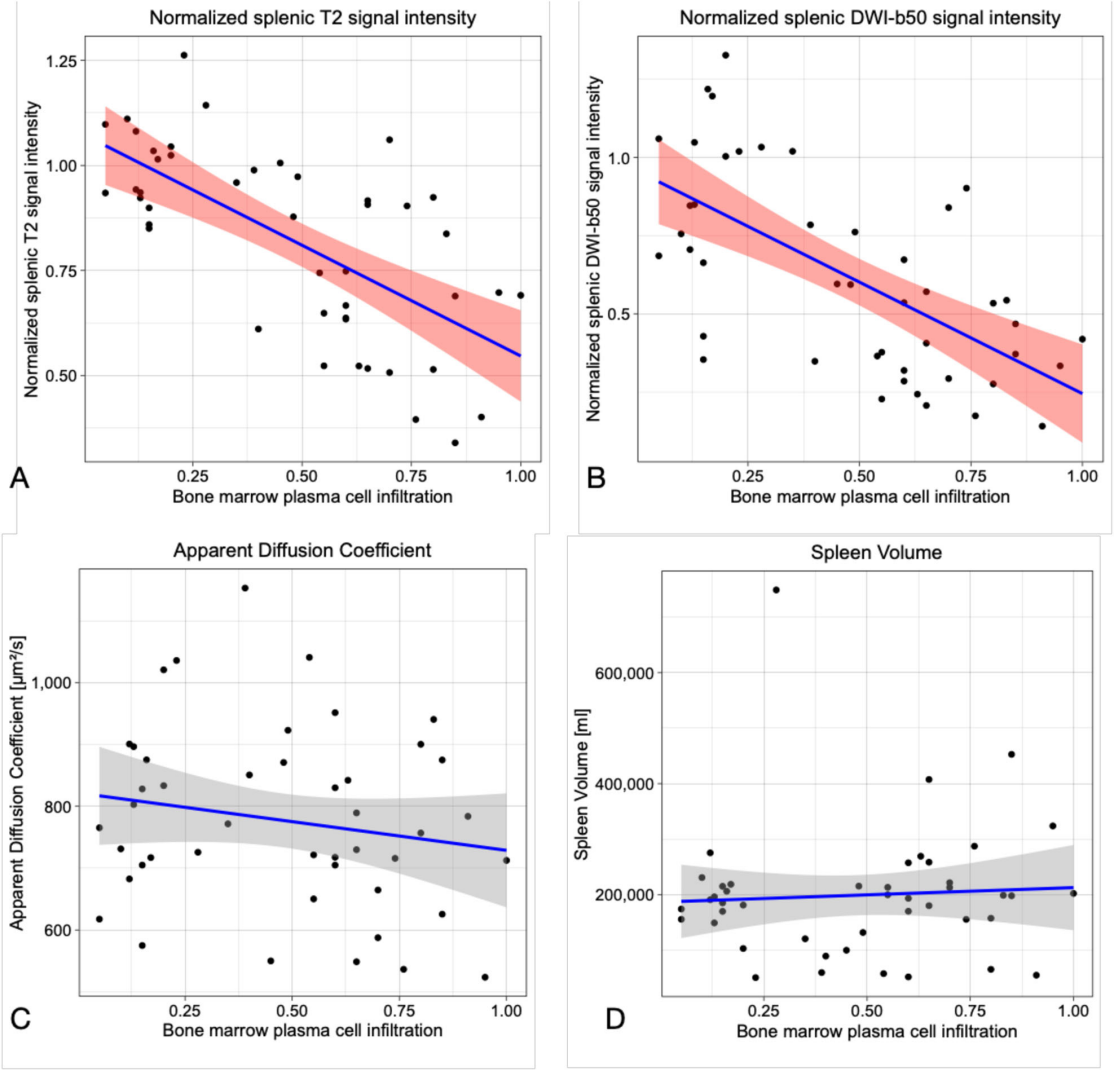
Scatter plots show a significant correlation of bone marrow plasma cell infiltration with normalized T2 signal intensity on T2-TSE-STIR (**A**) and b50 DWI (**B**); meanwhile, there is no significant correlation between plasma cell infiltration with apparent diffusion coefficient (**C**) or spleen volume (**D**)

Example MR images of two patients with low and high bone marrow plasma cell infiltration with and without loss of T2 spleen signal on DWI and T2-TSE are shown in Fig. 3. Scatter plots of bone marrow plasma cell infiltration with DWI, T2 signal intensity as well as ADC and splenic volume are shown in Fig. 4.

Furthermore, we identified a correlation between T2 spleen signal and markers of anemia, such as red blood count (*r* = 0.57; *p* < 0.001), hemoglobin (*r* = 0.48; *p* = 0.001) and hematocrit (*r* = 51; *p* < 0.001).

In the setting of identical scanner hardware and imaging parameters, image normalization did not significantly improve the correlations. After normalization, forming a spleen-to spinal cord ratio, absolute Pearson’s r increased slightly for mean splenic T2 signal intensity on DWI-b50 and T2-TSE images, while it decreased on normalized DWI-b800 (nT2: *r* = −0.66, *p* < 0.001; nb50: *r* = −0.64, *p* < 0.001; and nb800 *r* = −0.58, *p* < 0.001, respectively); however, the difference was not significant (T2: *p* = 0.27; b50: *p* = 0.26; b800 *p* = 0.33).

### Splenic FDG uptake on [^18^F]FDG PET-CT

In the subcohort of 39 patients who underwent concomitant PET-CT there was no significant correlation between splenic [^18^F]FDG uptake evaluated both as an absolute value (SUVmean, SUVmax) and in relation to tracer uptake in other organs which served as background (SLR, SBR) neither with bone marrow plasma cell infiltration on biopsy nor with splenic T2 signal (see Table 4).

**Table 4:** Pearson’s correlation of SUVmean and SUVmax spleen-to-liver and spleen-to-blood pool ratio with plasma cell infiltration and normalized splenic T2 signal intensity (Pearson's r [confidence interval], *p*-value)

	Plasma cell infiltration (PCI)	Normalized mean splenic T2 signal
Spleen-to-liver SUVmean ratio	0.24 [−0.08, 0.52]† *p* = 0.147	−0.13 [−0.47, 0.32]† *p* = 0.43
Spleen-to-liver SUVmax ratio	0.15 [−0.14, 0.46]† *p* = 0.365	−0.16 [−0.45, 0.17] *p* = 0.334
Spleen-to-blood pool SUVmean ratio	−0.01 [−0.26, 0.36]† *p* = 0.959	0.11 [−0.36, 0.45]† *p* = 0.52
Spleen-to-blood pool SUVmax ratio	0.11 [−0.18, 0.43]† *p* = 0.492	−0.01 [−0.43, 0.36]† *p* = 0.956

† A bias-corrected and accelerated bootstrap with 1000 samples was applied

## Discussion

The principal findings of this study can be summarized as follows: First, an elevated tumor load correlates with a decline in splenic T1, T2, and DWI signal intensities with a more pronounced effect observed on the intrinsically T2-weighted sequences, specifically T2-STIR and DWI. Second, this phenomenon is not associated with changes in spleen size, cellular density (quantified by ADC), or metabolic activity on FDG PET-CT.

Reported loss of spleen visualization or spleen signal might therefore be a result of decreased T2 signal intensity. This is because whole-body diffusion-weighted MRI (DWI) generally utilizes inherently T2-weighted spin-echo Echo Planar Imaging sequences with added diffusion-sensitizing gradients [23, 24].

There was no significant change in apparent diffusion coefficient (ADC), which is thought to represent isolated diffusion contribution and is a valuable measure for assessing cellularity in tumors [25].

In bone marrow optimized MR protocols, in MM, fat suppression using short TI inversion recovery (STIR) adds a T1 relaxation time contribution [26]. This might be an additional contributor as T1 signal intensity was also decreased. However, the effect was significantly lower.

The correlation between normalized splenic T2 signal intensity and plasma cell infiltration (*r* = −0.66) indicates a moderate to strong negative relationship with tumor burden [27]. Although the correlation strength was comparable to that of DWI (b-50) (*r* = −0.64), the ease of acquisition and the wider availability of T2-weighted imaging make it useful when DWI is unavailable or image quality is poor [28]. Future prospective studies should explore T2 or T2* mapping, which provides fully quantitative, objective measurements and would eliminate the need for normalization [29]. This may be particularly useful for longitudinal assessment of changes during therapy and relapse [30].

The underlying pathophysiology of this observed signal loss remains unclear. It has been proposed that loss of spleen visualization might be due to amyloidosis, direct plasma cell infiltration, or extramedullary hematopoiesis (EMH) [9, 10].

Splenic amyloidosis is known to decrease the T2 signal and there was a correlation between the T2 signal and myeloma protein [31–34]. The literature is less conclusive for T1 signal behavior. In contrast to other organs, some reports suggest increased splenic T1 signal intensity in amyloidosis [32, 34]. However, in a recent publication, Lama et al found no significant differences in splenic T1 and T2 times in patients with cardiac AL amyloidosis versus normal patients with a modified Look-Locker inversion recovery used in cardiac MRI [35]. Furthermore, no patient in our cohort had known systemic amyloidosis, as this was an exclusion criterion for the trial.

Instances of direct plasma cell infiltration of the spleen have been documented in MM [36, 37]. Despite these reports, we did not find significant ADC changes that would indicate hypercellularity nor was there an increased spleen size or increased metabolism on PET-CT. Notably, a recent study identified an inverse relationship between the tumor burden in MM and splenic uptake of [^68^Ga]Pentixafor, a specific marker for hematopoietic cells, including myeloma cells [38]. This observation implies a potential decrease in myeloid and lymphoid cell populations within the spleen, rather than an increase [38].

Our results confirm a link between markers of hematopoietic insufficiency (low Hb and complete blood count) and spleen signal. Rasche et al proposed extramedullary hematopoiesis (EMH) as a possible cause for the lack of signal, as they observed high splenic uptake in [99m]Tc-labelled anti-CD66 scintigraphy in one patient [9]. However, the specificity of CD66 (carcinoembryonic antigen) as a marker for EMH is limited. It is expressed not only on myeloid cells (including promyelocytes and granulocytes) but also frequently on MM plasma cells [39] and in several other malignancies [40]. Recently, Terrao et al presented a patient who showed a loss of spleen signal on DWI but no evidence of splenic EMH on pathological examination within 3 months. On the other hand, a patient with established EMH due to a myeloproliferative disorder and smouldering myeloma displayed a normal spleen signal [10].

We wish to put forth an alternative hypothesis suggesting the potential presence of iron accumulation within the spleen. This hypothesis is supported by the exceptional sensitivity of MRI in detecting iron deposition. The susceptibility effect stemming from this iron accumulation leads to a faster T1 and especially T2 decay with loss in signal intensity within the affected tissues [41]. Hepcidin, the primary regulator of iron homeostasis, is markedly elevated in multiple myeloma due to cytokines induced by the myeloma cells [42, 43]. Hepcidin blocks iron efflux from enterocytes and blood-degrading macrophages, particularly within the spleen [44]. This results in the accumulation of hemosiderin within macrophages and hampers iron mobilization, a central mechanism contributing to anemia in multiple myeloma [43, 45, 46]. Therefore, iron retention in the spleen could explain the signal loss in patients with high disease burden as well as the correlation with anemia. More generally, if this holds true imaging of the spleen could help to differentiate anemia of chronic disease from other causes (such as renal deficiency or replacement of the hematopoietic bone marrow). However, further studies are needed to confirm this hypothesis.

Our study had some limitations. Even though all our patients were scanned with the same hardware and sequence parameters, quantification of the T2 signal on relative T2 weighted images is limited. We addressed this problem with a normalization by calculating the spleen-to-spinal cord ratio as proposed by Terao et al [10]. A more objective alternative would be the direct quantification of T2 by T2-relaxometry [47], which should be pursued in future prospective studies.

Second, biopsies as the gold standard have well-recognized limitations for the measurement of plasma cell infiltration. Reasons include (1) sampling error because of uneven spatial distribution or patchy infiltration, (2) dilution of BM aspiration by peripheral blood, and (3) the lack of objectiveness in counting plasma cell proportion on BM trephine biopsy [7]. IMWG diagnostic criteria address this problem by defining bone marrow plasma cell infiltration (PCI) as the higher value of both bone marrow (BM) biopsy and BM aspiration [11]. However, quantification of the T2 signal of the spleen might improve quantification of the global tumor burden, especially when BM biopsy and aspiration are discrepant.

Similar to previous studies, direct pathological assessment of the spleen was not possible as spleen biopsies are rarely performed due to the risk of hemorrhagic complications [48]. Future animal studies could help bridge the gap between imaging findings and underlying pathology [49]. Finally, the retrospective design and our sample size of 45 patients may limit the generalizability of our findings. Future prospective studies with larger, multicenter cohorts and standardized protocols will be needed in order to validate our results and to establish splenic T2 as a clinical biomarker in MM.

In summary, our results indicate that the reported loss of spleen visualization on DWI is due to the loss of T2 signal. Hence, quantification of splenic signal intensity on T2-weighted imaging (not necessarily DWI) or by dedicated T2 relaxometry could be used as a surrogate marker for disease burden in patients with MM. Future studies are needed to explore the utility of T2 in longitudinal follow-up for evaluating treatment effects and prognosis.

## Supplementary information

ELECTRONIC SUPPLEMENTARY MATERIAL

## Abbreviations

[18 F]FDG: F18-fluorodeoxyglucose
ADC: Apparent diffusion coefficient
Dice score: Sørensen-dice coefficient
DWI: Diffusion-weighted imaging
EMH: Extramedullary hematopoiesis
ICC: Intraclass correlation coefficient
MM: Multiple myeloma
MRI: Magnetic resonance imaging
nb50: Normalized b50 diffusion-weighted imaging
nb800: Normalized b800 diffusion-weighted imaging
nT2: Normalized T2-TSE imaging
PET-CT: Positron emission tomography-computed tomography
SBR: Spleen-to-blood pool SUV ratio
SLR: Spleen-to-liver SUV ratio
STIR: Short TI inversion recovery
SUV: Standardized uptake value
T1-TSE: T1-weighted fast spin echo
T2-TSE: T2-weighted fast spin echo
VOI: Volume of interest
