# Supplementary material for: Splenic T2 signal intensity loss on MRI is associated with disease burden in multiple myeloma
Source: Eur Radiol. 2024 Nov 27;35(6):3576–86. doi: 10.1007/s00330-024-11191-8 (PMC12081551; doi:10.1007/s00330-024-11191-8)

Splenic T2 signal intensity loss on MRI is associated with disease burden in multiple myeloma

ELECTRONIC SUPPLEMENTARY MATERIAL

Supplement Table 1: Imaging parameters

	**T1-weighted TSE**	**T2-weighted TSE STIR**	**Diffusion-weighted imaging**
Sequence	2D TSE	2D TSE STIR	Diffusion-EPI iShim
Plane	coronal	coronal	axial
Slice thickness, distance factor	5.0mm, 10%	5.0mm, 10%	6.0mm, 0%
In-plane resolution (interpolation)	1.3mm x 1.3mm	0.7mm x 0.7mm (interpolated)	1.8mm x 1.8mm (interpolated)
FOV/acquisition matrix	375mmx500mm / 432x768	352mmx470mm / 360x640	287mmx460mm / 160x256
Number of stations (whole-body / pelvis)	5/1	5/1	5/2
Coverage in z-axis per station	375mm	353mm	228mm
Acquisition time per station	1min 43s	1min 7s	2min 49s
Parallel imaging	GRAPPA (Ac.-f. 2)	GRAPPA (Ac.-f. 3)	GRAPPA (Ac.-f. 2)
TR/TE/NSA	528ms/8.4ms/1	3650ms/56ms/1	5130ms/64ms/1
Flip angle	150°	140°	90°
Fat suppression technique	-	slice-selective IR (TI=160ms)	slice-selective IR (TI=180ms)
B-values	-	-	b values: 50/800 s mm^{-2}

Abbreviations: TSE: turbo spin echo; STIR: short-TI inversion recovery; DWI: diffusion-weighted imaging; EPI: echo-planar imaging; iShim: integrated slice-specific dynamic shimming; FOV: field of view; GRAPPA: Generalized Auto-calibrating Partially Parallel Acquisitions; Ac.-f.: acceleration factor. TR: repetition time; TE: echo time; NSA: number of signal averages; IR: inversion recovery, TI: inversion time.

Supplement Figure 1: Bland-Altman plots for mean T2-TSE and DWI (b50) signal intensity.

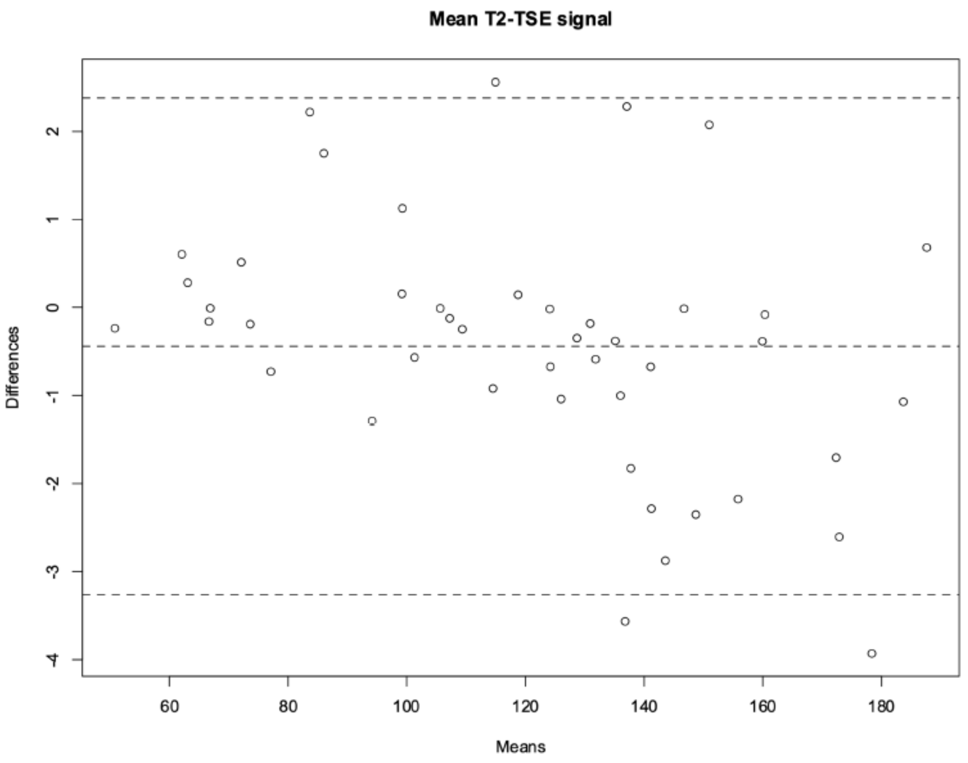

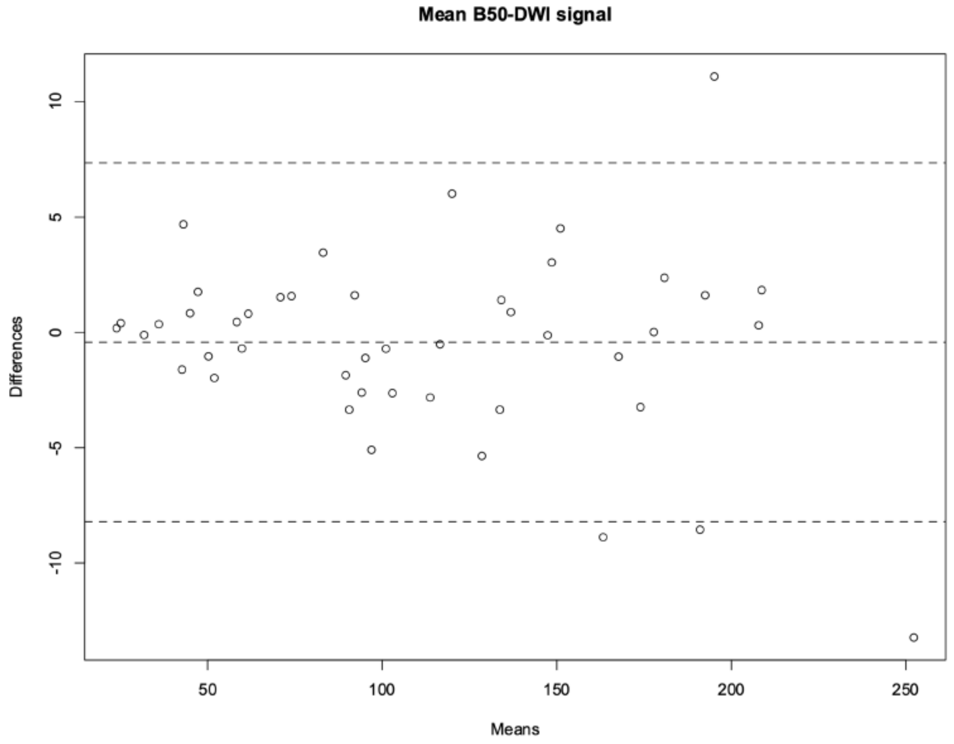

Supplement: Supplementary file 1 — ELECTRONIC SUPPLEMENTARY MATERIAL [file 330_2024_11191_MOESM1_ESM.pdf]
